# Toward deep learning replacement of gadolinium in neuro-oncology: A review of contrast-enhanced synthetic MRI

**DOI:** 10.3389/fnimg.2023.1055463

**Published:** 2023-01-23

**Authors:** Elisa Moya-Sáez, Rodrigo de Luis-García, Carlos Alberola-López

**Affiliations:** Laboratorio de Procesado de Imagen, ETSI Telecomunicación, Universidad de Valladolid, Valladolid, Spain

**Keywords:** gadolinium-based contrast agents (GBCAs), contrast enhancement, deep learning, synthesis, synthetic MRI, neuro-oncology, brain tumors

## Abstract

Gadolinium-based contrast agents (GBCAs) have become a crucial part of MRI acquisitions in neuro-oncology for the detection, characterization and monitoring of brain tumors. However, contrast-enhanced (CE) acquisitions not only raise safety concerns, but also lead to patient discomfort, the need of more skilled manpower and cost increase. Recently, several proposed deep learning works intend to reduce, or even eliminate, the need of GBCAs. This study reviews the published works related to the synthesis of CE images from low-dose and/or their native —non CE— counterparts. The data, type of neural network, and number of input modalities for each method are summarized as well as the evaluation methods. Based on this analysis, we discuss the main issues that these methods need to overcome in order to become suitable for their clinical usage. We also hypothesize some future trends that research on this topic may follow.

## 1. Introduction

Magnetic resonance imaging (MRI) is a non-invasive imaging technique widely used in clinical practice for the study of neuroanatomy. Gadolinium-based contrast agents (GBCAs) have become a crucial part of MRI acquisitions in the brain for the detection, characterization and monitoring of a wide range of diseases, such as multiple sclerosis (Silver et al., 1997), brain tumors (Zahra et al., 2007), and Alzheimer's disease (Khan et al., 2014; Montagne et al., 2016), among others. Indeed, ~40% of all MRI examinations in Europe and in the United States are performed with GBCAs (Runge, 2016). Particularly, for high-grade gliomas, where there is usually damage of the blood brain barrier (BBB), GBCAs are essential to improve lesion detection and monitoring due to the contrast enhancement visible in the T1-weighted images after the GBCA injection (Warntjes et al., 2018).

However, compared to the acquisitions without GBCAs (hereinafter referred to as native), the usage of GBCA in MRI results in patient discomfort during intravenous injection and increases the need of skilled manpower, hardware and thus, costs (Shankar et al., 2018). In addition, longer scan times in contrast-enhanced (CE) acquisitions reduce the accessibility to MR scans and might also lead to motion artifacts and, consequently, to extra efforts in re-acquiring or post-processing (Xie et al., 2022). Additionally, safety concerns have recently arisen due to the possible deposition of the GBCAs in the brain (Gulani et al., 2017), especially, in patients who need several follow-up acquisitions, as it is the case of oncological patients. Finally, GBCAs are known to be water pollutants, leading to environmental issues (Inoue et al., 2020). All of these issues encourage the avoidance of GBCAs in MRI routine exams.

Safety and environmental concerns of GBCAs could be tackled by the usage of new contrast materials (Wesolowski and Kaiser, 2016) or novel sequences in which the injection is not required, such as amide proton transfer (APT) imaging (Zhou et al., 2003) or arterial spin labeling (ASL) (Petersen et al., 2006). Nevertheless, these techniques, albeit promising, still require complex and expensive acquisition schemes. In addition, the resulting images are usually more difficult to analyze.

Deep learning (DL) could also be a potential solution for minimizing GBCA exams due to the impressive results that this technique has achieved in a wide range of image processing applications, such as reconstruction (Yang et al., 2017), segmentation (Akkus et al., 2017) and synthesis (Chartsias et al., 2017). Actually, the literature contains a growing corpus of DL works which aim at reducing, or even eliminating, the need of GBCAs in MRI; specifically, these works propose approaches for the synthesis of full-dose CE MRI from either low-dose or native acquisitions, using different network architectures.

In this paper we review the state of the art related to the synthesis of CE MR images that use DL techniques. Only journal papers are taken in consideration for the sake of brevity. In addition, we also analyze the evaluation methods used, current applications and possible future trends, as well as the main limitations that could hinder their clinical usage.

## 2. Deep learning synthesis methods

The DL methods included in this review were selected with a search within the well-known repositories Scopus and Web of Science (WOS). The search consisted in the Scopus query: TITLE-ABS-KEY[(~contrast enhanced~ OR ~post contrast~ OR gadolinium OR gbca* OR multimodal*) AND (synthesi* OR virtual) AND mri AND (~deep learning~) AND (~brain tumor*~ OR glioma OR glioblastoma OR *oncology)] AND LANGUAGE(english) AND SRCTYPE(j), and was adapted accordingly for WOS. The query retrieved 20 and 19 manuscripts in Scopus and WOS, respectively. After a first reading, eight out-of-scope manuscripts were discarded from the former repository and 10 from the latter. Finally, eliminating coincidences we ended up with a total of 12 manuscripts.

The methods that focus on the synthesis of CE weighted images could be divided into two main groups depending on the nature of the inputs (i.e., lose-dose vs. only native inputs). Both groups are discussed below, and a summary of the main characteristics of each of these methods can be found in Table 1.

**Table 1:** Summary of the DL synthesis works reviewed.

**Ref**.	**Inputs**	**Outputs**	**Dataset**	**#Scans** (Training^*^/Test)	**#Institutions**	**Vendors**	**#Scanners**	**Network**
**a) Decreasing gadolinium dose**								
Gong et al. (2018)	T1w and	ceT1w	30 mixed-conditions	60	1	GE	6	2D UNet
	ceT1w_10*%dose*_		and 30 glioma	(10/50)				
Pasumarthi et al. (2021)	T1w and	ceT1w	640 heterogeneous	640	3	GE, Siemens	8	2.5D UNet
	ceT1w_10*%dose*_			(69/571)		and Philips		
Luo et al. (2021)	T1w and	ceT1w	83 heterogeneous	83	1	Philips	1	2.5D UNet
	ceT1w_10*%dose*_			(30/53)				
Ammari et al. (2022)	T1w, T2w-FLAIR, ADC	ceT1w	145 primary brain tumors	145	1	GE	2	3D UNet
	and ceT1w_25*%dose*_		or brain metastases	(107/38)				
**b) Bypassing gadolinium injection**								
Kleesiek et al. (2019)	T1w, T2w, T2w-FLAIR,	ceT1w	82 healthy	116	–	Siemens	2	3D BayesUNet
	DWI (x3)^†^ and SWI (x4)^††^		or glioma	(104/12)				
Dai et al. (2020)^**^	T1w, T2w,	T1w, T2w,	274 glioma	274	BRATS2015	–	–	StarGAN
	T2w-FLAIR, ceT1w	T2w-FLAIR, ceT1w		(220/54)				
Sharma and Hamarneh (2020)^**^	T1w, T2w,	T1w, T2w,	285 glioma	285	BRATS2018	–	–	2D (Multi-Modal) GAN
	T2w-FLAIR, ceT1w	T2w-FLAIR, ceT1w		(270/15)				
Hu et al. (2021)^**^	T1w, T2w,	T1w, T2w,	800 glioma	800	BRATS2017	>1	>1	UNet + CNN
	T2w-FLAIR, ceT1w	T2w-FLAIR, ceT1w		(533/267)	+ TCGA + 1		
Preetha et al. (2021)	T1w, T2w, T2w-FLAIR,	ceT1w	2061 glioblastoma	6929	230	–	–	3D UNet or
	and (optionally) ADC			(5005/1924)				3D CGAN
Chen et al. (2022)	T1w, T2w, and ADC	ceT1w	300 brain tumors	426	1	Siemens	1	3D CNN
				(411/15)				
Xie et al. (2022)	T1w	ceT1w	369 glioma	369	BRATS2020	–	–	3D Retina UNet +
				(200/169)				Synthesis module
Wang et al. (2022)	T2w-FLAIR	ceT2w-FLAIR	221 unkown	221	2	GE, Siemens	4	2D-to-3D GAN +
				(148/73)		and Canon		3DGAN (synthesis)

a) Methods that propose the synthesis of full-dose CE images from their low-dose counterparts. b) Methods that propose the synthesis of full-dose CE images from only native images. Note that the dash indicates the absence of that datum in the manuscript. The image modalities are T1-weighted (T1w), CE T1w (ceT1w), T2-weighted (T2w) fluid-attenuated inversion recovery (T2w-FLAIR), CE T2w-FLAIR (ceT2w-FLAIR), apparent diffusion coefficient (ADC), diffusion weighted imaging (DWI), and susceptibility weighted imaging (SWI).

* Training value refers to the summation of training and validation data for the manuscripts in which these two values are given separately.

** Multimodal method: it allows the synthesis with different configuration of inputs and outputs. Contrast-enhanced T1w image modality could be among the outputs.

† Diffusion weighted imaging (DWI) group: *b* = 0, 1,200 mm^2^/s and the ADC map.

†† Susceptibility weighted imaging (SWI) group: SWI, magnitude SWI, phase SWI, and a minimum intensity projection SWI.

### 2.1. Decreasing the dose of gadolinium

Methods that focus on the reduction of GBCAs range from 25% of the dose (Ammari et al., 2022) to 10% of the dose (Gong et al., 2018; Luo et al., 2021; Pasumarthi et al., 2021). As a reference, 100% of the dose typically corresponds to 0.1 mm/kg. As can be seen in Table 1a, all of these methods use as input the native T1-weighted (T1w) and the low-dose CE T1w (ceT1w). In Ammari et al. (2022), the T2-weighted (T2w) FLAIR (T2w-FLAIR) and the apparent diffusion coefficient (ADC) are also considered as inputs. The main difference between Gong et al. (2018) and Pasumarthi et al. (2021) is the size of the dataset employed, being considerably higher in the latter.

Regarding network architectures, all of the authors used a convolutional neural network (CNN) and, specifically, a UNet (Ronneberger et al., 2015) which includes a contracting path that encodes the input into a set of feature maps followed by a (symmetrical) expansive path that decodes these features. Some differences could be found in the data dimensionality processed by the networks (i.e., 2D slices or 3D volumes). Notice that Luo et al. (2021) and Pasumarthi et al. (2021) use a 2.5D network because the network processes the data by chunks of 7 slices to avoid inconsistent image enhancement across slices.

### 2.2. Bypassing gadolinium injection

A complete bypass of the GBCA injection is sought by other authors; their methods are summarized in Table 1b. While earlier methods used several input modalities to compensate for the lack of GBCA-related information in the inputs, a trend for reduction of the number of inputs has been since observed. Actually, the last two entries in the table (Wang et al., 2022; Xie et al., 2022) report the synthesis from only one input modality; to this end, in Xie et al. (2022), a more sophisticated synthesis method is used, which consists in two steps, namely, a 3D Retina UNet module and a synthesis module. UNet architectures are also common among these methods (Kleesiek et al., 2019; Hu et al., 2021; Preetha et al., 2021; Xie et al., 2022), but other authors opt for different configurations of generative adversarial networks (GANs) (Dai et al., 2020; Sharma and Hamarneh, 2020; Preetha et al., 2021; Wang et al., 2022). Still, the UNet is widely used as the backbone (i.e., generator network) of the GANs as in Dai et al. (2020), Sharma and Hamarneh (2020), and Wang et al. (2022). Interestingly, methods in this section use larger datasets than those in Section 2.1.

### 2.3. Discussion about the methods

One of the main questions about the synthesis of CE weighted images is the number and the relevance of the required input modalities. First, the inclusion need of a low-dose CE image should be assessed. Assessing whether native image modalities have all the information required for a truthful prediction may be controversial. For example, in Bône et al. (2021) — a previous conference paper of the same authors than Ammari et al. (2022), see Table 1a—, the authors identified that the low-dose ceT1w was key for the synthesis, and the performance of the synthesis method systematically and significantly dropped when the low-dose image was not provided as input. However, the authors referred to in Table 1b achieved high quality synthesis only with native image modalities as inputs, at the expense of larger datasets. Note that in some methods such as Gong et al. (2018) and Pasumarthi et al. (2021) the testing sets are larger than the training sets, which is an unusual practice for which the authors do not provide a clear explanation. The former conducted a quantitative evaluation of the model on a separate dataset of gliomas to test if performance varied for a specific clinical indication, and the latter focused on achieving a balanced training set by including approximately an equal number of studies from all the institutions and scanner manufacturers. These two aspects, together with the fact that both studies performed data augmentation to enlarge the size of the training subset, may be the reasons behind these decisions.

Network architecture and loss function also have important implications for the quality of synthesized images. In Preetha et al. (2021) performance significantly improved when using a CGAN as opposed to a UNet. In addition, the loss function has been tailored in the different works to improve the overall synthesis quality: Pasumarthi et al. (2021) introduced a combination of weighted L1 loss, adversarial loss, and perceptual loss, while Chen et al. (2022) weighted the tumor regions; Xie et al. (2022) incorporated the tumor contours as prior knowledge to focus on the contrast enhanced lesions. On the other side, Wang et al. (2022) proposed atrous spatial pyramid pooling (Chen et al., 2017), improved residual blocks and deep supervision for better lesion location and anatomical and texture detailed synthesis.

Additionally, some experiments have also been conducted to find out the importance of each native input. Chen et al. (2022) showed that the usage of T1w as the only input obtains a satisfactory performance, albeit the inclusion of T2w and ADC provides an improvement, with the former modality proving more relevant for the synthesis than the latter. These results seem approximately in line with those reported by Preetha et al. (2021), who found that including ADC in addition to native anatomical images (i.e., T1w, T2w and T2w-FLAIR) did not increase performance. In this case, they found that T1w was the most relevant image, followed by T2w-FLAIR and T2w. It is true that in Chen et al. (2022) the inclusion of ADC did slightly improve performance, but it could be caused by the fact that T2w-FLAIR was not used in that work. Nevertheless, Kleesiek et al. (2019) obtained completely different results since they found that T2w followed by the DWI group (*b* = 0, 1,200 mm^2^/s and ADC) were the most influential image modalities for the synthesis. Yet, these results do not fully agree with Bône et al. (2021), who found that T2w-FLAIR, followed by ADC and T1w was the order of modality removal with less impact in the quality of the synthesized images. These questions need further research until a consensus is reached.

In contrast, some works follow a complete different approach and propose multimodal methods (Dai et al., 2020; Sharma and Hamarneh, 2020; Hu et al., 2021) in which the input and output modalities are not fixed. Thus, they can deal with a variety of synthesis combinations including the synthesis of ceT1w images. The synthesis of different CE image modalities simultaneously is an interesting—and challenging—application of CE synthetic MRI. However, we are not aware of any published paper that pursues this sort of contrast-enhanced joint synthesis.

## 3. Evaluation

The quality of the synthesized images needs to be assessed in order to ensure their actual utility. These evaluations are commonly accomplished in terms of quantitative and qualitative analyses. Some authors also study the utility of the synthesized images in different clinical applications.

### 3.1. Image quality metrics

Most of the authors (Gong et al., 2018; Kleesiek et al., 2019; Dai et al., 2020; Sharma and Hamarneh, 2020; Pasumarthi et al., 2021; Preetha et al., 2021; Ammari et al., 2022; Chen et al., 2022; Wang et al., 2022; Xie et al., 2022) perform a quantitative and nonsubjective analysis of the synthesized images. Metrics commonly used are peak signal-to-noise ratio (PSNR) and structural similarity index (SSIM). Whereas PSNR measures voxelwise differences between acquired and synthesized images, SSIM measures nonlocal structural similarity between both. These metrics are computed between the acquired and the synthesized CE images.

Additionally, some authors use other metrics such as mean square error (MSE) (Sharma and Hamarneh, 2020), mean absolute error (MAE) (Wang et al., 2022), normalized MAE (NMAE) (Dai et al., 2020; Xie et al., 2022), Pearson correlation coefficient (PCC) (Xie et al., 2022), visual information fidelity (VIF) (Dai et al., 2020), naturalness image quality evaluator (NIQE) (Dai et al., 2020), and area under the curve (AUC) of the receiver operating characteristic (ROC) curve (Kleesiek et al., 2019; Ammari et al., 2022; Wang et al., 2022).

### 3.2. Reader studies

Subjective visual quality of the synthesized CE images is also usually assessed by means of qualitative analysis with board-certified neuroradiologists. Different reader studies are considered: in Gong et al. (2018), readers were asked to evaluate the synthesized CE images with 5-point Likert scales (1, poor; 5, excellent) regarding image quality, suppression of aliasing/motion artifacts, and the enhancement degree compared to native images. Also a 5-point scale was employed for the readers in Ammari et al. (2022) to evaluate image quality. Qualitative assessment including image quality and lesion detection, enhancement, and conspicuity, were performed in Luo et al. (2021). In Kleesiek et al. (2019), the authors performed a Turing Test; readers were asked whether they could distinguish between acquired and synthesized CE images. Finally, in Pasumarthi et al. (2021) readers were asked to classify each image regarding the presence of enhanced and non-enhanced structures, and the effect of vessel conspicuity on clinical diagnosis was also studied.

### 3.3. Clinical applications

To evaluate the impact that these methods could have in clinical practice, whether quantitative algorithms can reliably work with these synthesized images needs to be tested and the value of these images for improving clinical decision making should also be quantified.

#### 3.3.1. Tumor segmentation

Quantitative tumor segmentation is one application used to validate the agreement between acquired and synthesized CE images. To this end, Pasumarthi et al. (2021) automatically segment the tumor core with a DL method (Myronenko, 2018) using only the CE images. Segmentations were carried out twice, once on the acquired CE image and then on the synthesized image. The average segmentation Dice score between the acquired and the synthesized images was 0.88 ± 0.06 with a median value of 0.91, and the tumor segmentation masks showed good visual agreement. Preetha et al. (2021) also perform two CE tumor segmentations but using a multimodal input to the DL segmentation method (Kickingereder et al., 2019) consisting of native T1w, T2w, T2w-FLAIR, and ceT1w; in each segmentation either the acquired or the synthesized ceT1w was used along with the other three native images. The results shows a median underestimation of 7% of the tumor volume as compared with acquired ceT1w, but, in general, a good correlation between both. However, this multimodal segmentation approach could hide the actual implications of the synthesis in the segmentation, since an overall aggregated effect is measured.

#### 3.3.2. Tumor response assessment

In Preetha et al. (2021) the authors performed a volumetric tumor response assessment in patients with different follow-up exams. First, they computed the CE tumor volume on both the acquired and the synthesized ceT1w images. Next, they assessed the time to progression by analyzing the longitudinal change in tumor volumes for both cases. A median time to progression of 4.2 months (95% CI 4.1–5.2) was reported with the synthesized ceT1w, whereas with the acquired ceT1w they obtained a median time to progression of 4.3 months (4.1–5.5). Finally, the two values of the time to progression were employed as surrogate endpoints for predicting the patient's overall survival with time-dependent Cox regression models. Using the data derived from the synthesized ceT1w, the hazard ratio predicted was 1.749 (95% CI 1.282–2.387, *p* = 0.0004), which is similar to the 1.799 (95% CI 1.314–2.464, *p* = 0.0003) obtained with the data derived from the acquired ceT1w.

## 4. Limitations

The synthesized CE images present, overall, a good visual resemblance and high quality metrics. Actually, the reader study in Ammari et al. (2022) showed how the synthesized images were preferred over the acquired by the radiologists in some cases. However, the image smoothness and the network failures in the synthesis of small structures, with the small vessel and the small-sized lesions being especially challenging cases, are common problems found by most of the authors.

Except multimodal methods (Dai et al., 2020; Sharma and Hamarneh, 2020; Hu et al., 2021), the rest of them can only deal with the scenario in which both input and output image modalities are fixed; however, this is not the typical case in the clinical routine. Thus, ideally, models should be able to adapt to variations of images modalities. Specifically, the requirement of a large amount of different image modalities as input could be especially problematic, since this availability could be limited in real-world practice. For example, in Kleesiek et al. (2019) as many as 10 input channels are required for a correct synthesis. In addition, all the input images should be perfectly registered, and this is not only laborious but could also lead to potential synthesis inaccuracies. Indeed, misregistration of input images is a recurrent problem across the methods. This registration failure creates fake intensities outside the region of actual enhancements which could have implications in the diagnosis. In contrast, the implicit motion-artifact correction and aliasing suppression are upsides of these methods.

Other methods carry out the synthesis from only a few native image modalities (Dai et al., 2020; Sharma and Hamarneh, 2020; Chen et al., 2022) or even from only one native input (Xie et al., 2022). However, these methods were solely validated with image quality metrics (SSIM, PSNR, etc.), which might not fully represent the actual utility of the synthesized images in quantitative clinical applications. Thus, more validation is required in order to assess their true clinical value for diagnosis.

Although there are some exceptions such as Pasumarthi et al. (2021) and Preetha et al. (2021), who have validated their methods with large multi-institution, multi-vendor datasets, generally speaking the proposed approaches need to be validated with larger datasets including acquisitions from different institutions, vendors, and scanner characteristics in order to ensure their robustness and reproducibility. In addition, the trained networks will probably not be able to synthesize those lesions or abnormalities that were not included in the training dataset. Thus, for the sake of generalization, the training dataset should be as heterogeneous as possible.

Finally, common to most DL synthesis methods, the lack of interpretability is also an important limitation which could hinder their clinical usability. Having a model that could self-explain which are the most informative parts of the input images for the synthesis would allow us to get further insight into the image generation procedure.

## 5. Future trends

In clinical practice it is customary that contrast enhancement is estimated by subjective visual comparison between native T1w and ceT1w images or by using rules of thumb such as full width at half maximum for tumor or scar extension. This is because the intensity of weighted images has an arbitrary scale. However, parametric maps are known to have an absolute scale and to be more robust against scanner imperfections, which are relevant ingredients for them to qualify as biomarkers of tumor burden. Indeed, some studies show how parametric maps might provide means for accurate identification and quantification of contrast-enhanced regions (Blystad et al., 2020; Pirkl et al., 2021). Hence, combining synthetic MRI approaches with Quantitative MR may be a promising next step in order to quantify the enhancement without the need of GBCAs.

Recently, we have proposed a self-supervised synthetic MRI approach for the computation of T1, T2, and PD parametric maps and the synthesis of non-acquired weighted images from only two acquired weighted images (Moya-Sáez et al., 2022). Self-supervised learning allowed us to compute the parametric maps without the need of reference parametric maps for network training.

Following this method we have performed an experiment aimed at quantifying the reliability of this synthesis methodology; specifically, starting with the data available in Moya-Sáez et al. (2022), we have modified the self-supervised network there described in order to perform the synthesis from only the T1w modality. This way, we can train the network with only the T1w images and the T1, T2, and PD parametric maps could be generated from only that input. The training was performed with the same leave-one-out scheme and hyper-parameter setting as in Moya-Sáez et al. (2022). Afterwards, in production mode (i.e., once the network is trained), a ceT1w image is input to the network. Under the assumption that the only difference between both images (native T1w and ceT1w) is the GBCA intake, the resulting maps should correspond to the CE parametric maps. In this case, the subtraction between the T1 map and the ceT1 map should give us a quantification of the enhancement map.

**Figure 1:**
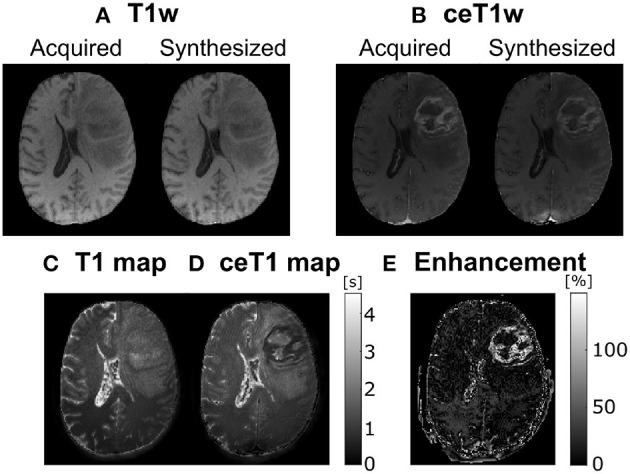
A representative axial slice of parametric maps computed by the self-supervised CNN and the synthesized images. **(A)** Acquired and corresponding synthesized native T1w from the computed T1, T2, and PD maps. **(B)** Acquired and corresponding synthesized contrast-enhanced T1w (ceT1w) from the contrast-enhanced T1 (ceT1), T2, and PD maps. **(C)** native T1 map. **(D)** ceT1 map. **(E)** Corresponding enhancement map computed as the subtraction between **(C)** and **(D)**. T1 and ceT1 maps are measured in seconds (s), whereas the enhancement map is measured in (%).

Experiment results are shown in Figure 1, in which we can observe a representative slice of the resulting synthesized weighted images out of the computed parametric maps for both the native and the ceT1w images—figures (A) and (B) respectively—used as input; these synthesized weighted images [right hand side of figures (A) and (B)] can be visually compared with the actually acquired counterparts (left hand side) and visual resemblance is noticeable. In addition, Figure 1C shows the T1 and ceT1 maps computed by the self-supervised CNN—and used to calculate the synthesized images in figures (A) and (B)—. Finally, figure (E) shows the enhancement map computed as the normalized difference between the T1 and ceT1 maps, specifically, $100*\frac{\left|\text{T1}-\text{ceT1}\right|}{\text{ceT1}}\left[\%\right]$. For the sake of visibility the enhancement map intensity is cropped between 10 and 150%. Interestingly, the reduction of the T1 values within the enhanced region in the enhancement maps can be noticed.

In terms of architectures, the onset of solutions based on attention mechanisms may give rise to new actors in the scene that could leverage the synthesis quality. Some interesting activity has been carried out in the image synthesis field using attention gates (Liu et al., 2022) and transformers (Dalmaz et al., 2022), although not applied to CE synthesis in neuro-oncology. The former proposes the synthesis of ceT1w from native T1w, albeit it is applied to brain aging of healthy subjects and Alzheimer's disease patients with remarkable results; the latter focuses on the synthesis of native weighted images, but the synthesis of CE weighted images is not considered.

## 6. Conclusion

The synthesis of CE weighted images could have high impact on clinical practice, not only by reducing costs and shortening protocols, but also by alleviating safety concerns about GBCAs usage. Several DL methods have been recently proposed for the synthesis of CE images from low-dose or even from only native images, with promising outcomes. In the future, the confluence of Synthetic MRI and Quantitative MRI could be a keystone toward automated diagnosis and prognosis in neuro-oncology.

## Author contributions

EM-S, RL-G, and CA-L contributed to the conception and design of the study. EM-S reviewed the papers. EM-S and CA-L wrote the first draft of the manuscript. All authors contributed to manuscript revision, read, and approved the submitted version.
